# Transactions of the Alumni Association

**Published:** 1877-09

**Authors:** 


### Transactions of the Alumni Association.

The Transactions of the Association of the Alumni and Officers of the Medical Department of the University of Buffalo, will be issued in a few days.

The volume will make a handsome book of about one hundred and fifty pages, printed on tinted paper, in plain type. It will contain the Addresses delivered at St. James Hall before the Association by Drs. F. H. Hamilton, Sanford Hunt and W. W. Potter; the papers read at the Alumni Meetings; the addresses of the Presidents; the poems, and a synopsis of the proceedings at the banquets. Every alumnus of the Buffalo Medical College should have a copy. The price has been fixed as follows: In paper covers, fifty cents; in boards, one dollar. Those desiring copies should remit to Dr. E. N. Brush, 65 Ellicott St,, Buffalo, N. Y., who will acknowledge all receipts at once, and forward the volume as soon as issued. As the printing is in an advanced state of completion, it is expected that the work will be ready for subscribers by the first week in September.

